# The relevance of transversal competences in vocational education and training: a bibliometric analysis

**DOI:** 10.1186/s40461-020-00100-0

**Published:** 2020-11-06

**Authors:** Inmaculada Calero López, Beatriz Rodríguez-López

**Affiliations:** Universidad Nacional de Educación a Distancia (UNED), Madrid, Spain

**Keywords:** Transversal competences, Soft skills, Employability, Vocational education and training

## Abstract

Vocational Education and Training (VET) programmes have included the acquisition of transversal competences in their curricula as a tool to increase employability. The number of researches has exponentially grown in the last years, emphasizing its relevance and the multiple approaches and factors involved in the learning process. The present bibliometric study aims to provide an overview of the scientific research carried out during the last 10 years and to shed some light on several relevant topics in this field. The results indicate the need to improve students’ transversal competences in order to meet the demands of the labour market, the importance of the collaboration of all the actors involved in the process (policy makers, industry and educators) and from a pedagogical point of view, the necessity of introducing new teaching approaches to implement and assess the acquisition of transversal competences. However, and despite the surge of interest in the study of transversal competences in the last decade, further empirical research is needed, especially at Vocational Education and Training level, to understand how transversal competences develop and what kind of initiatives have an impact of their acquisition.

## Introduction

Vocational Education and Training (VET) is a key element of lifelong learning systems that aims to equip people with the technical knowledge, expertise, skills and competences required on the labour market and, at the same time, with the personal skills for their future lives in society. It is the tool to pave the way towards high-quality jobs and increased employability. Despite its importance, in many countries participation in VET has traditionally been stigmatized in favor of university studies, although the recent socio-economic transformations are changing attitudes toward VET (Aldossari 2020).

Educational institutions at European and national levels agree on the idea that all programmes of study should give students the chance to connect academic learning with the areas of knowledge and skills needed both for professional and for private lives and enable them to become lifelong learners (Fung 2017; Rychen 2016). At the same time, globalization and technological changes are transforming the needs of employers, who are now looking for candidates with demonstrated transversal competences or soft skills (adaptability, leadership, teamwork, clear communication in different languages, etc.…). This new scenario has made transversal competences a common topic in the research field. Nevertheless, while some studies are revealing a convergence between the competences included in educational programs and the employers’ perspectives (Oria 2013), others show that the relevance of the different transversal competences may be perceived differently by the different actors engaged in the educational process (Sá and Serpa 2018; Renold et al. 2018). Even more, some studies published recently (European Commission 2018; INEE 2019; OIE 2017) conclude that students perceive that their academic studies are not preparing them well for work. This debate on how to combine knowledge with skills concerns educators as well, since they are responsible for finding the way to empower, motivate and engage their students (Greenberg and Nilssen 2015) and redefine their methodologies and curricular plans accordingly.

As stated before, globalisation is creating an increasingly diverse and interconnected world and this new society requires mastering socio-cultural tools for interacting with others. Transversal competences related to the capacity individuals have to communicate, take initiative, work in a team or solve problems are among the requirements that the new organization of work demands (Bañeres and Conesa 2017). Furthermore, Cinque (2016) highlighted that “emotional intelligence studies also support the hypothesis that interpersonal skills are more likely to predict successful careers and that they are necessary for the increasing use of teams, the rapid pace of globalization, the capacity to dialogue in a cross-cultural environment, and the growing need to retain talent in organizations” (401). Thus, training programs must teach these competences alongside the specific or technical ones, to prepare students for functioning well in society and in the workplace (OECD 2005; European Commission 2018).

Education is changing as a response to the new demands and so is the role of teachers. Nowadays, educators must adopt a motivating role, analyse the implications of the learning process and introduce new methodologies that help students to acquire the necessary skills for becoming lifelong learners. According to González-Peiteado and Rodríguez-López (2017), understanding how our students acquire knowledge is key for activating the learning process. However, we must not forget the role of policymakers and their interest in learning from the best examples of VET systems. VET systems are structured differently in different countries in terms of objectives, implementation of transversal competences and the relationship between education and the labour market. In 2016, Pilz, taking a multi-perspective approach, proposed a new typology of VET systems using six different countries as case studies. The results show important differences but also similarities among the countries. “The typology offers both a framework for further explanatory approaches in individual country contexts and an opportunity for international comparison of key aspects of VET systems, such as the value attached to vocational qualifications and the possible transfer of VET models from one country to another” (Pilz 2016, 295). Those countries with strong VET systems have demonstrated their capacity to attract talent, increase enrolment and decrease youth unemployment together with a rapid adaptation to the technological changes and the ability to match labour market demands and this is partly due to the incorporation of transversal competences in their curriculum and the balance between the theoretical and practical contents. The research on the transferability of these successful VET systems to other countries has also been subject of study with varying results (Renold et al. 2018; Baumeler 2019; Euler 2013; Graf et al. 2014; Alemán 2015).

To sum up, the number of researches on transversal competences has exponentially grown in the last years, emphasizing the relevance of the topic at different levels and for all the actors in the education process. VET programmes have included transversal competences in their curriculum convinced of their capacity to boost students’ employability. For teachers, understanding employers and students’ perceptions of relevant transversal competences and how they develop will allow them to adapt their methodology and contents to students’ needs and consequently, to contribute to their successful entrance in the labour market.

Bearing in mind the above, the present bibliometric study is aimed, on the one hand, at gaining insight into the growing interest and relevance of including transversal competences as part of the VET curriculum, and on the other hand, at discussing the main implications of the research conducted so far in the field and at identifying those competences that have a positive influence on the student’s overall education and therefore aid their integration into the workforce.

## Method

The current study arises from a retrospective ex-post facto design (Montero and León 2002) through a thematic and bibliometric analysis. The purpose of this study is to provide an overview of the scientific research conducted on transversal competences over the past ten years at Vocational and Educational Training level. We do not claim that this is an exhaustive all-inclusive collection, but a selection of the most relevant studies published in the field during the last decade.

A collection of papers, mainly empirical studies, published between 2010 and 2019 was used to construct the dataset for this study. To this end, three different searches have been conducted in three international databases: EBook Education Collection (EBSCO), Education Resource Information Center (ERIC) and Web of Science (WOS). First, several keywords searches were conducted involving permutations of relevant terms (i.e. ‘competences’, ‘soft skills’, ‘vocational education’ and ‘VET’). The Boolean equation was SU transversal competenc* OR SU soft skill AND TI vocational education or TI VET NOT TI Higher Education. The first search gave 2304 results in EBSCO, 9341 results in ERIC and 3338 results in WOS. Then, to carry out a selection of the research studies to be included, the following advanced criteria were established: studies ranging from 2010 to 2019, published in English and Spanish, quantitative studies, primary and secondary research studies, whose focal point, for the sample population, had to be VET and the topic had to be related to soft skills or transversal competences and the full text available. After applying these limiters, and once duplicated articles were excluded the number of studies were reduced to 188.

Once we got the list of 188 documents, subsequent screening sequences (type of document, intended audience and educational level) were applied and the results reviewed and limited to the aim of our study. By type of document, we keep only empirical research published in books, journal articles or doctoral dissertations. Encyclopedias, manuals, and anthologies were excluded because such edited volumes provide general overviews rather than research results. We limited our intended audience to researchers, policymakers, and teachers and excluded all the documents devoted to research on Higher Education and not VET. Thus, out of 188 articles initially selected, a total of 34 were chosen for the analysis. The remaining documents did not conform to our specific topic, within the scope of Vocational Education and Training, or they proved to be reports for information purposes or theoretical analysis.

### Categorisation of data

The information from the 34 publications were encoded in a spreadsheet based on the following categories:

- *Publication*: Every document was identified by the name of the publisher in order to understand the scope of interest in transversal competences. The dataset was broken down into four different categories:
  (i) International journals indexed by different databases such as SCOPUS, CrossRef or Academic Research Index (e.g. Int. Journal of Education Development, Int. Journal of Vocational Education and Training).
  (ii) Journals specialized in the field of professional, vocational and technical education indexed by ESCI, SCOPUS, SCImago, EBSCO (e.g. Empirical Research in Vocational Education and Training; Vocations and Learning; Journal of Vocational Education and Training, etc.).
  (iii) Journals with a more localised focus (e.g. Universities, Asia–Pacific Education Researcher, etc.) that present the research conducted in a specific geographical area.
  (iv) Other publications. In this group, we have included a PhD thesis dissertation, and several high-level conference proceedings and reports commissioned by education institutions and national centres on education.
- *Geographic distribution of the research*: The empirical research in our dataset were conducted in 21 countries and to extract some conclusions, the documents were grouped by continent.
- *Publication date and author(s):* The amount of research output is presented broken down by year.
- *Topic:* The documents are sorted into three central topics: (i) Achievement and assessment of transversal competences; (ii) transversal competences and employability; and (iii) implementation of transversal competences in VET models.

The table also includes the author(s) name(s) and three extra columns: one with the title of the article and two complementary columns where the main objectives and study results were summarised.

## Results

The initial and continuous training of transversal competences is central to the necessary preparation of future professionals (Sá and Serpa 2018) and this article provides an overview of the scientific research carried out during the last decade in relation to transversal competences in Vocational Education and Training students. The results are analysed in two different sections: the first one includes the analysis of the bibliometric data, and the second one analyzes the content results.

### Bibliometric analysis

**Fig. 1:**
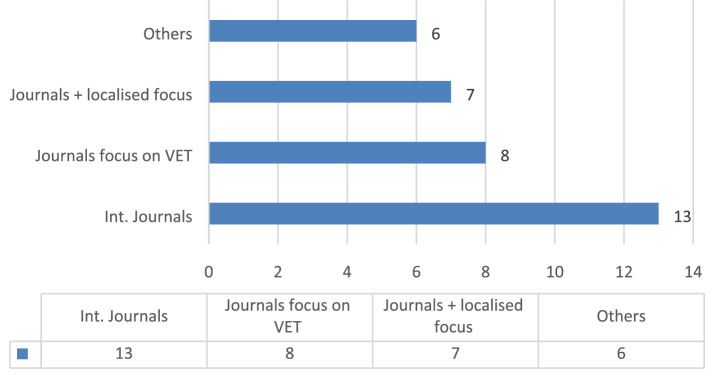
Type of publication
  The results showed a diverse distribution with a higher frequency of international journals, which confirms the importance of the topic at an international level. These international journals aim to report new insight and foster critical debate about the role that education plays in the development of society and to share the results of empirical research which can be implemented in any technical and vocational school and in the workplace. The next group encompasses specialized journals devoted to empirical research in the field of professional, vocational and technical education. They explore topics such as the effectiveness of different vocational education systems at the school, business and policy-maker levels, pedagogical practices, apprenticeship and competence development in vocational education and training. The third group includes publications of empirical studies in education, with emphasis on the experiences of successful educational systems in a specific geographical area, and the last group includes a PhD thesis dissertation and some conference proceedings and educational reports. A further point of interest about the diversification of data is the corroboration of the relevance of the transversal competences for the different actors involved in the education and employment systems and both at international and national level.

The 34 selected documents were classified based on the following categories:

- *Publication*: The dataset was broken down into four different categories:
  International journals: 13 articles
  Journals with a focus on the educational level: 8 articles
  Journals with a more localised focus: 7 articles
  Other publications: 1 PhD Thesis dissertation, 2 conference proceedings and 3 reports commissioned by education institutions and national centres on education (Fig. 1).

*Geographic distribution of the research*: Our data revealed a diverse distribution. The empirical research in our dataset were conducted in 21 countries. To extract some conclusions, the documents were grouped by continent. Thus, out of 34 documents, 15 (44.12%) were produced in Europe, 8 (23.53%) in Asia, 3 (8.82%) in Africa, 2 (5.88%) in North America, 1 (2.94%) in Oceania and 5 (14.70%) presented data from different areas. From the data we learnt, that there is an increased interest in Europe and Asia to study and understand better the development of transversal competences, and a need to conduct more intensive research in Africa, an area where more of the underdeveloped countries are situated. The fact that almost 15% of the research is conducted by cooperation among different countries supports the idea of the interest in learning from the experience of the most advanced countries and the extend of research on transferring the successful VET programs to the areas where the system is not so well implemented yet.

**Fig. 2:**
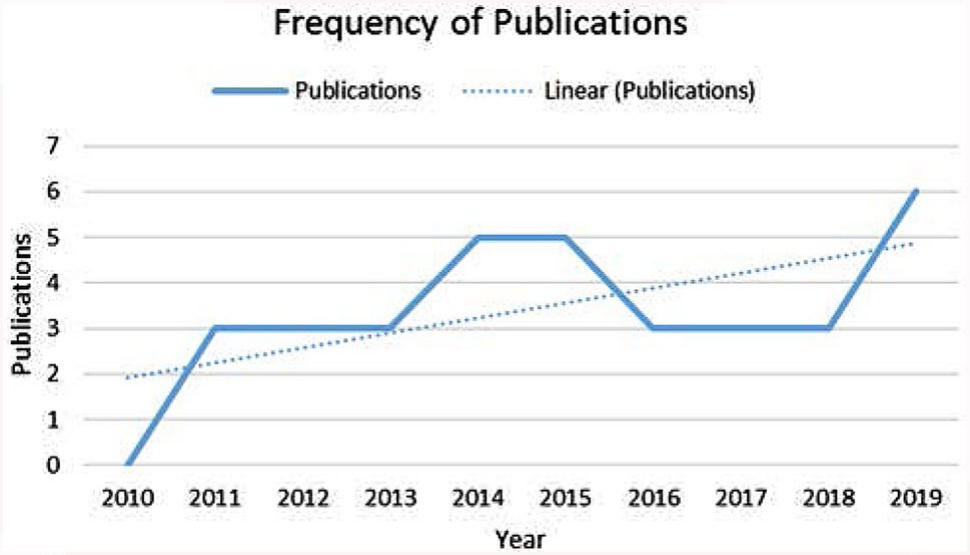
Frequency of publications on transversal competences 2010–2019

*Publication date:* It presents the amount of research output broken down by year. Based on our sample (n = 34), there has been a steady overall growth trajectory, with the final period covered by the analysis (2019) including double as many studies as the first. This reflects a remarkable increase in the level of research activity and the growing interest in transversal competences in VET systems (Fig. 2).

### Content analysis

To analyse the articles, we used a thematic review approach to reflect and synthesize the main research topics. After a close reading, we identified three main research topics: a) the assessment and achievement of transversal competences, b) the relationship between the acquisition of transversal competences and employability and c) the description and implementation of transversal competences in VET models.

Table 1 presents the three research topics, the number of articles (n) dealing with each topic and the author(s) and year of publication.

**Table 1:** Articles analysed by topic

Topic	n	Author(s) (year)
Assessment and Achievement	13	Ab Rahman et al. (2014); Abdullah-Al-Mamun (2012); Adnan et al. (2014); Baartman and Ruijs (2011); Baumeler (2019); Fjellström (2014); Lahn and Nore (2019); Kyllonen (2013); Misbah et al. (2019); Monnier et al. (2016); Munastiwi (2015); Sturing et al. (2011); Rahman (2011)
Employability in VET students	10	Asonitou (2019); Boahin and Hofman (2013); Boyer (2017); Gekara and Snel (2018); Hasanefendic et al. (2016); Moldovan (2019); Oviawe et al. (2017); París et al. (2018); Rajadurai et al. (2018); Tukundane et al. (2015)
Implementation of transversal competences in VET models	11	Alemán Falcón (2015); De Bruijn (2012); Euler (2013); Graf et al. (2014); Järvi (2012); Nugraha et al. (2016); Powel and Fortwengel (2014); Renold et al. (2018); Stapa et al. (2015); van Griethuijsen et al. (2019); Yang (2015)

### Assessment and achievement of transversal competences in VET students

Although all the articles included in this section focused on the achievement and assessment of transversal competences at Vocational Education and Training level, we have identified three different points of view, which at the same time are interconnected: the students’ perspective, the educators’ approach and the policy makers and stakeholder's viewpoint.

According to Baartman and Ruijs (2011), students’ perceived competence relates to metacognition and self-efficacy and there is a reciprocal relationship between self-efficacy as a perception and academic performance as an objective measure. Success in academic performance will lead to a strong sense of self-efficacy and self-efficacy will lead to an increased probability of success. According to the authors, when the perceived competence is evaluated from the students’ perspective, the results show a decrease in their perceived competence during the year when students start with their internships. “This might be due to the fact that students get a more accurate picture of the complexity of the workplace and can compare their own competence to the requirements of the workplace and the competence level of other more experienced employees" (Baartman and Ruijs 2011, 395).

Baartman and Ruijs (2011) suggest that in the future, curricula should focus more on realistic students' perceptions of their competence (e.g. by self-assessment and reflection) to foster self-confidence in students and propose as future lines of research the analysis of the effects of different educational programmes in perceived competence (e.g. programmes including internships from Year 1). They also highlight the need to conduct a longitudinal research on individual students' development process to understand how components of competence develop in relation to each other and how they integrate towards a more holistic professional competence.

The lack of empirical evidence of competence-based vocational education (CBVE) impact on students’ competence development was the base of Misbah, Gulikers and Mulder’s study (2019). Briefly, we can say that the concept of competence-based education is twofold (Nederstigt and Mulder 2012). On the one hand, the construction of the well-qualified professional, that is, an individual who possesses the competences needed in the current labour market. On the other hand, the concept that competence-based education will endow people with certain personal, soft, and methodological competencies (being entrepreneurial, innovative, reflective, critical, and motivated for lifelong learning) which allow them to be capable of adapting to future challenges. Specifically, in this study, they compared competence and knowledge development of students in several vocational schools in Indonesia that have implemented principles of CBVE to a higher or lesser degree. The findings showed a higher competence development in those students attending high-CBVE than those attending low-CBVE, corroborating the positive effects of the implementation of CBVE.

In his study, Fjellström (2014) describes and analyses students’ perceptions of vocational competence and identifies how this competence is built up. The result indicates a gap between acquired vocational competence in practice and the related learning goals in the course syllabuses. The study concludes that the developed vocational competence fits better the demands of the industry than the course goals and that the project complexity also affects the students’ motivation, autonomy and their ability to develop the required competences. Therefore, task complexity must be adapted to students’ level. If the task is too difficult, students will be demotivated but if it is too easy, the students will not be challenged enough to develop vocational competences.

However, Adnan et al. (2014) address the students’ perspective differently and try to find out which soft skills are relevant for them and their perception of preparedness to use them in the workplace. The study results reveal that, although the students feel that soft skills are essential to succeed in their job career, most of the participants need to enhance and develop some of them, especially those related to lifelong learning. The authors propose the modification of the existing teaching–learning strategies to integrate soft skills more successfully in the curriculum. Similarly, in a study on the development of generic skills among technical students in Malaysia, Rahman et al. (2011) stated the need to improve students’ transversal competences and proposed that educational authorities should identify the gaps in the current curriculum to provide a richer learning environment and to train teachers accordingly to the new needs.

The idea of integrating soft skills in the curriculum leads us to the second group of studies, those centered on educators’ role. Education should generate learning environments that promote lifelong learning (European Commission 2019; Council of the European Union 2018) and educators play a strategic position in managing the learning process in formal education. They are responsible for designing and managing the process that will prepare their students to be competitive in the labour market (Munastiwi 2015). According to Munastiwi (2015), quality assurance is an important aspect to improve in education in order to anticipate problems. He proposes five elements to be considered in the implementation of the quality assurance model: guidelines, policies, targets, management mechanisms, and activities. In this new scheme, VET teachers are requested to be creative and to integrate “the learning plan, the local potential, teaching materials, and instructional media” (Munastiwi 2015, 224) to guide and support VET students and boost the acquisition of transversal competences.

We have seen that classrooms can be a place to practice with alternative ways of facilitating learning and integrating hard and soft skills in a formal educational context. However, soft skills seem to be difficult to teach and even harder to assess in that context (Abdullah-Al-Mamun 2012). Whereas some programmes for international student assessment have a limited focus on students’ performance or generic competencies (e.g. Programme for International Student Assessment (PISA)), for vocational skills, student assessment should take into account a large variety of vocational domains and important differences in the educational systems between countries. In addition, the development of assessment tools for VET competences cannot be based on extensive previous research and accumulated knowledge about vocational expertise (Muja et al. 2019) because so far, there are few empirical studies conducted at this level. Hence, the design of such tools should be tested for feasibility and an agreement needs to be reached on the definition of concepts such as “vocational competence” or “generic competence” (Lahn and Nore 2019). Other experts consider that these competences have to be defined and assessed in an occupation-specific way. That is the case of Monnier et al. (2016), who developed a simulation-based situational judgment test for medical assistants in Germany. The results show that the competence levels are satisfactory for all dimensions, which confirms the need to integrate these skills in the curriculum.

VET system has adopted in many countries a competence-based education and training (CBET) methodology which makes it necessary to identify what forms of assessments must be undertaken to meet its demands (Ab Rahman et al. 2014; Lahn and Nore 2019). In other words, the current challenge is to set a valid, reliable and practical way to assess the acquisition of those competences in VET. In Ab Rahman et al. study (2014), competence assessment is divided into performance (practical activity) and objective (tests) assessment, giving more value to the practical activities than to the theoretical ones. Nevertheless, the lack of evaluation standards complicates the evaluation of student competency. The authors affirm that “to get good quality and accurate assessment of evaluation there is a need to develop analytical standard reference criteria for teachers and students as well” (Ab Rahman et al. 2014, 1075).

Sturing et al. (2011), taking into account previous studies (Wesselink et al. 2007 cited in Sturing et al. 2011), developed a model of comprehensive competence-based education (CCBE model) to provide study programme teams working in vocational education with an instrument to assess to which extend their programmes were based on the principles of CBE. In their article, they present the results of two studies based on teachers’ feedback. The aim was to identify the necessary modifications to make the CCBE model a valid instrument for assessing the level of compliance with the CBE. The results were positive and they showed that “teachers understood and interpreted the revised model as intended, were able to position their study programmes by using the revised model and that the content validity of the revised model was good” (Sturing et al. 2011, 191).

In the twentieth century, theoretical knowledge was considered the most important determinant of educational and workforce outcomes and this led to an extensive reliance on test scores for university admission and employment screening. In the twenty-first century, the experts recognize that soft skills (motivation, teamwork, effective communication, etc.) play an important role in determining success in the school and the workplace. The way soft skills evolve and the role of education in developing those skills, inevitably lead to the development of new education, training and assessment methods (Kyllonen 2013).

The acquisition of transversal competences is considered as a key element for effective employment and an essential part of the role of VET as well. To produce individuals equipped with the adequate skills adapted to the demands of the social, political and economic situation, VET institutions need to work together with the industry and policymakers. Nowadays, countries with well-developed VET systems present lower youth unemployment rates (Baumeler 2019), therefore, policymakers seek to borrow this model from those countries to transfer to their own countries. Baumeler (2019), taking as an example the dual VET system in Switzerland, explores the question of whether this successful concept can be transferred to a completely different context, as it is the case of India. The results show that the pedagogical concept of competence-based is not transferable due to the different “culturally coined educational concepts and practices in the context of schooling” (Baumeler 2019, 11). We will discuss this further in t*he implementation of transversal competences in VET models*’ section and in our conclusions.

### Transversal competences and employability in VET students

The current economic situation makes no longer enough for graduates to have academic or technical knowledge, but they are also required to develop those skills used at the workplace, which ultimately empower them as lifelong learners. In this context, employability, seen as having the skills, knowledge and attitude required to get a job, is gaining importance. However, while the introduction of new Dual VET systems combining theoretical curriculum with internships has progressed, the acquisition of employability competences is still hampered by a curriculum that according to employers and the students themselves prevents or, at least, does not adequately encourage the development of transversal competences (OIE 2017; Sá and Serpa 2018; Boahin and Hofman 2018). The following studies investigate from different perspectives transversal competences in terms of competency gaps and employers’ demands.

Rapid changes in the economy and the labour market can create imbalances between VET programmes and industry needs because it becomes more difficult to anticipate future skill needs in the workplace. There is an ongoing debate about whether employability skills can be developed within or outside the educational context, but there is agreement on the fact that different interrelated factors (e.g. students’ characteristics, training instructions, cooperation with industry and government educational policies) are determinant for their successful acquisition. Based on these assumptions, Boahin and Hoffman (2013) developed a conceptual model to explain the potential influence of various factors on the acquisition of employability skills. The results confirmed significant relationships between academic disciplines and the acquisition of employability skills and emphasized the need for teachers to adopt a more student-centred approach, and to introduce authentic work experience that enhances students’ autonomy and creativity. Teamwork is considered to promote the acquisition of other soft skills (communication, collaboration, problem-solving, leadership, etc.) and emerges as the dominant skill across all the disciplines analysed.

Frequently, employers complain that graduates who enter the job market today are not equipped with the right set of soft skills that would enable them to integrate and contribute effectively at the workplace (Abdullah-Al-Mamun 2012) and some experts have explored this issue. For example, Asonitou and Hassall (2019) examined the incorporation of professional skills into Greek accounting education studies. The results indicated, on the one hand, that all stakeholder groups considered professional skills as important, but there were some differences in the importance assigned to relevant skills, and in how the educational process should develop them. On the other hand, the study shows the necessity of introducing new teaching approaches and improving the critical thinking abilities of students “through the development of a curriculum that supports graduates’ employability and sustainable development” (Asonitou and Hassall 2019, 1), and this opens the way to further investigation on how transversal competences can be taught and incorporated in VET curricula.

This is not the only study on the analysis of the existing knowledge and skills gaps and the relevance of the acquisition of transversal competences for employability. Rajadurai et al. (2018) investigated the gap between the knowledge, skills, abilities and personality of technical students and their actual performance in employment as assessed by a group of Human Resource managers. The results demonstrated the importance of developing transversal competences to enhance graduates’ competency, reliability, creativity and flexibility to meet industry requirements. Another example is Moldovan’s (2019) study included in the Erasmus + project iNduce 4.0. He conducted a survey among small to mid-size enterprises (SME) and VET providers from six countries. The objectives were to present an up-to-date analysis on the knowledge and skills gaps on the topic of Industry 4.0, to define the main difficulties in organizing work-based training according to SME and to create a work-based learning (WBL) methodology for VET students and apprentices in order to boost “SMEs’ capacity for transition by nurturing the necessary skills for the ‘factory of the future’” (Moldovan 2019, 295).

A different approach on the incorporation of soft skills in educational programs and its relationship with employability is Boyer’s study (2017) on the benefits of participating in English for Speakers of Other Languages (ESOL) classes integrated with training for vocational skills. The objective was to determine whether the integration of English language instruction with a vocational program influenced participants’ job-seeking activities and employability. The main findings and recommendations of the study were, first, that developing a curriculum requires a multi-layered approach including training, education, and career development. Second, the participants were aware of their learning needs and created learning strategies. And third, that “due to the complexity of learning needs in the current and future workforce, adult education and human resource development must work together to provide unique programs that will contribute to the learning needs of individuals, organizations, and society simultaneously” (Boyer 2017, 3). Hence, the conclusion is that the success of an educational program does not rely on a single factor but on the combination of different ones and the implication of all the actors in the educational process.

Similarly, in developing countries, educational exclusion and consequently, the lack of skills often leads young people to marginalization and unemployment. A VET system is expected to offer students some skills to ameliorate this situation. In Uganda, an explanatory study conducted on four VET programmes examined the current practices, and the youth preparedness for entering the labour market (Tukundane et al. 2015). Although the findings showed that VET could improve access to labour market, several aspects such as the negative social perceptions of VET, the teacher-centred methodology, the incompetently trained teachers lacking pedagogical training and motivation, or the inadequate facilities required improvement. Another example is a study conducted in Nigeria by Oviawe, Uwameiye, and Uddin (2017). As one of the developing countries in the world, Nigeria fights to bridge the existing gap between labour market needs and work force skills in collaboration with the industry. In their conclusions, the authors propose different future recommendations to improve the acquisition of competences in VET students, such as for the Nigerian government and the VET policymakers, the establishment of effective linkages with work market and for VET institutions, adequate monitoring, supervision and encouragement of internships.

An important aspect related to employability and transversal competences is the need to identify professional profiles and describe the functions, professional activities and relevant transversal competences for each of them. These were the main objectives of a study conducted in Continuing Vocational Education and Training (CVET) in Spain by París, Tejada and Coiduras (2018). Based on the results, three professional profiles were defined (training manager, trainer and mentor) with their respective functions, professional activities and specific and transversal competences. The authors concluded that the delimitation of the occupational profiles prevents professional intrusion, provides guarantees for quality professional work, and establishes levels of qualification, certification and accreditation to recruit competent professionals.

As the job market evolves, soft skills are becoming more and more important for VET graduates and job seekers. Many governments and educational institutions, aware of this situation, are working on redesigning their training systems to strengthen transferable soft skills. A study conducted in Australia (Gekara and Snell 2018) examines the challenges of developing transferable skills among Australia´s workforce and the conflicts that can emerge between different actors. VET systems aim to enable workers for significant flexibility in occupational mobility. Nevertheless, evidence suggests that many workers, particularly at the lower skill levels, find it difficult to use their existing skills to find employment in a different occupation (Sheldon and Thornthwaite 2005; Snell, Gekara, and Gatt 2016 cited in Gekara and Snell 2018). The results also show that the redesigned Australian VET system can equip the nation’s workforce with transferable skills, which would not only benefit employers but also allow workers to find employment across many occupations in an increasingly unstable labour market.

Globalization, which implies the interchange of worldviews, cultures, and ideas, has had a dramatic impact on education and on labour market. The education and training of the labour force are facing new challenges and the education institutions are increasingly demanded to provide the new workforce with the adequate knowledge and skills. The scarcity of skilled workers has often been attributed to the gap between educational systems and companies’ needs or to the fact that learning and training profiles are not suitable for current industry settings (Tijdens et al. 2012 cited in Hasanefendic, Heitor, and Horta 2016). A cross-national comparative case study in Portugal, Netherlands and Germany suggested that “strengthening problem-based learning and short-term project-oriented research through technical and vocational higher education can facilitate the process of training the work-force in skills of increasing relevance to local markets” (Hasanefendic, Heitor, and Horta 2016, 329), especially if training is made in collaboration among VET practitioners, institutions and local and economic actors.

### Implementation of transversal competences in VET models

VET is a major policy topic for countries all over the world. Policymakers want to know how strong VET systems deal with issues like rapid technological change, matching labor market demand for skills, attracting enrollment, and creating high-status VET programs. Although at first glance, the answer might seem simple – incorporating STEM subjects and the acquisition of soft skills in the curricula- the comparison of different VET programs has shown different results. “What differentiates the strongest and weakest VET programs is the level of linkage between actors from the education and employment systems.” (Renold et al. 2018, 1). To measure the education-employment linkage throughout all processes involved in a VET program, Renold et al. (2018) designed a tool (KOF Education-Employment) and compared VET systems in 20 countries. The results showed that countries with a dual VET system, which engages employers, education institutions and students through all processes, obtained the highest scores, while those with school-based VET programs had the lowest ones.

Dual VET programs, based on the German VET model, combine elements of the school-based learning with work-based practice. Their main objective is to produce skilled workers with flexible qualifications, capable of working in their chosen fields and to adapt to new job opportunities (Alemán 2015; Euler 2013). In practical terms, this means that the educational institutions and employers, always in compliance with the education policy, work together when designing training curricula. Students are trained in the school but also in company. Thus, they acquire theoretical knowledge and transversal competences that allow them to become lifelong learners and to adapt to the changeable work market needs. (Graf et al. 2014 Powell and Fortwengel 2014).

It is often suggested that the German dual VET system should be transferred to other countries. Among its benefits, many experts claim that this would stimulate economic growth in the importing countries and contribute to the reduction of youth unemployment rate (Alemán 2015; Euler 2013; Graf et al. 2014). Nevertheless, so far, the results have been disappointing. Despite the considerable effort from some governments, the results show that importing a system, involves more than mere duplication.

It is a process of selecting and adapting certain components to suit the objectives and conditions of the potential importing country. In the case of a vocational training system, a country seeking to reform its existing system does not simply replace it with that of Germany or any other country. Instead, it reviews the experiences of various countries and selects the features that best fit its own goals, structures and culture, adapting them as necessary (Euler 2013, 6).

According to Baumeler (2019), studies that investigated the transfer of the dual VET model to other countries came to the conclusion that only certain elements might be successfully implemented and the model needed to be adapted to each country. (Euler 2013; Fortwengel and Jackson 2016; Graf et al. 2014; Powell and Fortwengel 2014). In her case, the study case of the implementation of dual VET system in India, research results indicated, for example, difficulties in transferring elements of the socioeconomic context (i.e. different requirements from the labour market) and with regard to instructional aspects and aligned with competence-based VET (i.e. the ideal proactive, entrepreneurial, innovative and critical person, or the accompanying behavior of teachers and students).

In addition, the way we work today is completely different from the past. A global competitive environment, new management processes and new technologies that require employees to show autonomy, problem solving skills and a high degree of flexibility to adapt to the new circumstances and new technologies, characterize it. The current VET students have been in contact with new technologies (smartphones, tablets, video consoles) from an early age, so most of them have previous experience and prefer to use social media apps, text messages and blogs to interact and get information rather than the more traditional methods. Furthermore, blogs and social networks are highly motivating to students and very effective for collaboration and discussion (Jamaliah, Rohana and Aede Hatib 2012; Gialamas, Nikolopoulou, and Koutromanos 2013; Sandars dan Murray 2009 cited in Stapa et al. 2015).

Stapa et al. (2015) consider that Technical and Vocational Education (TVE) can be combined with e-learning using web-based applications to transfer knowledge in a theoretical and practical form and as a way to improve class attendance and participation. Based on the results of previous research, they state that “career excellence vocational education will be achieved through work-based learning oriented training programs by allowing the content knowledge and skills available to students and constantly being adjusted to conform to the new requirements in the industry, business and society” (Stapa et al. 2015, 128). According to the authors, factors such as engagement, attitude, and base knowledge are associated with the skill achievement of vocational students and present in e-learning. Nevertheless, e-learning strategies are still not being explored extensively in VET system. There is an ample scope for research in the area of the use of web applications (Facebook, Twitter, Instagram) and collaborative tools (Teams) in the participatory process of learning and teaching.

Certain researchers support the idea of exploring and implementing new methodological approaches to tackle the lack of engagement of many VET students. On the one hand, Yang (2015) proposed and evaluated a blended approach “with enhanced instructor support and scaffolding, provision of appropriate learning aids, and the use of collaborative learning” (Yang 2015, 290). In general, blended learning combines several features of face-to-face learning with the use of information technology in the form of e-learning strategies. This new approach for creating educational programs can take into account the individual differences between students and bring together different learning methods (Stapa et al. 2015). Results showed that this methodology could promote thinking skills (creative, critical and problem solving) and had a positive impact on academic achievement (Yang 2015).

Other experts propose the adoption of a Competence-based education (CBE). CBE refers to the integration of knowledge, skills and attitudes in the educational programme. Furthermore, CBE calls for authentic and functional learning, that is, the learning context, knowledge and the tasks included in the curriculum must resemble a real working environment. “CBE also calls for more personalised education (with a teacher functioning as an expert, coach, or mentor), for greater flexibility in educational programs, for self-assessment, and for more formative assessment. Students are also required to reflect on their own learning process and increasingly steer their own learning” (van Griethuijsen et al. 2019, 3). Van de Griethuijsen et al. (2019) investigated the effects that the level of Competence-based Education (CBE) implementation had on student satisfaction and the influence of CBE implementation in the relationship between teacher team-learning activities and student satisfaction. The results revealed that teacher team-learning was positively associated with the implementation of CBE and that CBE had a positive effect on student satisfaction, so it was concluded that the implementation of CBE had fulfilled its goal of better preparing students to enter the work market and that teacher team-learning could support the further implementation of CBE.

Nevertheless, CBE also has an impact on teachers’ role and teaching practice in VET (De Bruijn, 2012; Järvi, 2012). De Bruijn (2012) examined the teaching practices, related personal ideas and professional attitudes of ten teachers from five vocational schools to understand the interaction between teachers and students. Observation of teaching behaviour showed that teachers were working in the multifunctional environments required for establishing powerful learning environments and they tried to extend the authenticity of the learning environment. However, there was less variation in activities, learning material and learning contexts, in particular, when students were working and learning in a simulated environment.

One last example on the relationship between the VET system and the acquisition of soft skills is Nugraha et al. (2016) study. The study had a twofold aim: determining the students’ soft skills before and after the implementation of collaborative techniques and measuring the correlations between school type (Public with A grades, Private with A grades and Private without A grades), the students’ outcomes and the development of soft skills. The results confirmed an improvement of soft skills among students when cooperative learning was incorporated into their lessons and the lack of interaction between learning model and school type on the students’ soft skills.

## Conclusion and discussion

The present bibliometric study provides an overview of the scientific research carried out during the last 10 years in the field of transversal competences in VET. Despite the substantial studies published on transversal competences and stated their importance in achieving national and international education goals, there is a lack of reliable comparative data at vocational education and training level. After analyzing our search results, we can state that most of the publications were qualitative analysis or theoretical reviews, but not quantitative research based on empirical facts. Furthermore, we have not found any longitudinal study at our educational level, so we stated the need to carry out more research to understand how the transversal competences develop and what kind of initiatives have an impact of the acquisition of transversal competences.

Regarding the research topic of the *assessment and achievement of transversal competences in VET students*, we consider that students’ self-reports of their own competency levels are important for generating insights into the effectiveness of CBVE for fostering students’ competence development, but incomplete. Thus, we agree with those researchers who suggest combining self-reports with external sources, such as teacher reports (Baartman and Ruijs 2011). Because perceptions of students and teachers on student achievement might differ, considering both perspectives to analyse CBVE provides valuable and useful information to identify and strengthen its effectiveness.

One of the main challenges for researchers in this field is to define the relevant practices that influence students’ achievement in formal learning contexts. According to the experts, promoting self-confidence in students is linked with a higher probability of success (Baartman and Ruijs 2011). However, so far, more research is needed on realistic students’ perceptions of their competence and the analysis of the effects of different educational programmes in students’ perceived competence. At the same time, studying individual students' development process will help researchers to understand how components of competence develop in relation to each other and how they integrate towards a more holistic professional competence. Then, in the future, program designers and vocational educators should develop teaching models in which knowledge and competence development can be fostered in balance.

The second research topic is *transversal competences and employability in VET students.* Rapid changes in the economy and the labour market can create imbalances between VET programmes and industry needs, because it becomes more and more difficult to anticipate future skill needs in the workplace. Our search brought to light the scarcity of studies at VET level and the need to understand the job market’s requirements for our students. If we think about the COVID-19 sanitary and economic crisis, we understand the relevance of addressing the pressing issue of youth unemployment and the development of those competences which will allow the future employees to adapt to new situations. Educators have had to put in practice new ways of online teaching in record time with the main purpose of fostering autonomous learning and the development of the transversal competences most demanded by the job market at the moment. The so called “new normal” has revealed the need to boost online learning and the use of new technologies as tools to acquire the skills to face the current needs of companies that are adapting at breakneck pace to telework environments, online sales, remote customer service, etc.. In this new normal, employees must possess higher skills in organization, leadership, adaptability, autonomy, teamwork and communication, among others.

Besides, given the fact that the research on acquisition of transversal competences has highlighted the significant relation between the former and employability, particularly for young people entering the labour market (Cinque 2016), we consider the relationship between employability and soft skills to be worth investigating further, with the aim of identifying the most demanded skills by employers, and hence the most useful for the students’ preparation. Consequently, we will be able to put forward or adapt our courses to encourage these skills.

Finally, in relation to the *implementation of transversal competences in VET models*, we would like to highlight that nowadays, countries with well-developed VET systems present lower youth unemployment and policymakers are exploring the possibility to transfer this successful model to their countries. There is a general perception that the key for success lies in the curricula of VET programs and the combination of theoretical knowledge with internships. Nevertheless, we agree with Renold et al. (2018) in stating that the difference is on the level of relationship among all the actors in education and employment systems, emphasizing the need for collaboration among the different stakeholders to adapt these models to the real situation in each country.

We agree with the idea that internships are a key factor for integration in the work market. Internships allow students to apply the theoretical knowledge acquired in class, to develop and reinforce the professional skills in the workplace and they also offer the possibility of demonstrating their abilities and skills to entrepreneurs. For employers, the internships offer the opportunity to check if the future employees' competences are adapted to those required in the job (Mareque and De Prada 2018; Mareque et al. 2018). Nevertheless, we would like to highlight the importance of taking into account factors such as the duration of the internships. An adequate duration contributes to increasing levels of interaction and integration and can have a positive effect on the performance of functions (Coll and Eames 2004), there is no much research on this aspect so far, but steps need to be taken to analyse the duration and quality of the internships and their relationship with the acquisition of transversal competences.

In the light of the above, and even if the research on transversal competences in VET has exponentially grown in the last years, there is still a long way to go and many research fields and approaches to explore. Our initial objective was to focus our study only on transversal competences at VET, but the results showed that despite the interest of the topic at a theoretical level, there is not an extensive research body. The relevance of this article lies on two central aspects: proving the lack of empirical research in the field at VET level so far and thus, the need to conduct more empirical research to understand how transversal competences develop and what kind of initiatives have an impact of their acquisition. And presenting the most common topics analysed until now, shedding some light on the results and practical implications and proposing new lines of research for the future.

## Data Availability

The datasets used and/or analysed during the current study are available from the corresponding author on reasonable request.

## Abbreviations

CBE: Competence-based education
CBET: Competence-based education and training
CCBE model: Comprehensive competence-based education model
CBVE: Competence-based vocational education
CVET: Continuing Vocational Education and Training
EBSCO: EBook Education Collection
ERIC: Education Resource Information Center
ESOL: English for Speakers of Other Languages
KOF: Konjunkturforschungsstelle
PISA: Programme for International Student Assessment
SME: Small to mid-size enterprises
STEM: Science, technology, engineering and mathematics
TVE: Technical and Vocational Education
VET: Vocational Education and Training
WBL: Work-based learning
WOS: Web of Science
